# A multimodal scanning electron microscopy–atomic force microscopy images for early cervical cancer detection using discrete wavelet transform-derived features and divergence-based classification

**DOI:** 10.1590/1806-9282.20251173

**Published:** 2026-04-20

**Authors:** Gürkan Özbey, Sevcan Aytaç

**Affiliations:** 1 Adıyaman University, Department of Obstetrics and Gynecology – Adıyaman, Turkey.; 2 Firat University, Department of Electronic Technology – Elazığ, Turkey.

**Keywords:** Microscopy, atomic force, electron, scanning, Fluorescence imaging, Wavelet analysis, Information theory, Statistical distributions

## Abstract

**OBJECTIVE:**

The aim of this study was to enhance the early diagnosis of cervical cancer by utilizing both scanning atomic force microscopy and scanning electron microscopy images. Until now, these two imaging modalities have not been combined for this purpose. Relying on a single imaging technique may reduce diagnostic reliability. Therefore, a multimodal approach was developed to provide more accurate and consistent results.

**METHODS:**

A total of 540 scanning electron microscopy and 540 atomic force microscopy cell images were obtained from the Faculty of Nanotechnology and Scanning Electron Laboratories. The discrete wavelet transform method was applied to extract ideal feature regions from the images. At least three divergence-based classifiers, triangle divergence, Jensen–Shannon divergence, and Hellinger divergence, were employed to identify cervical cancer–related patterns. To improve classification reliability, feature likelihoods were calculated using exponential graph regulation. Feature weights were determined and incorporated into a combined classification function. Based on this, scanning electron microscopy and atomic force microscopy cells were categorized into six groups: normal scanning electron microscopy, normal atomic force microscopy, benign scanning electron microscopy, benign atomic force microscopy, and malignant scanning electron microscopy, malignant atomic force microscopy.

**RESULTS:**

The classifiers demonstrated interrelated performance, with triangle divergence achieving superior accuracy compared to Jensen–Shannon divergence and Hellinger divergence. The weighted feature approach improved diagnostic precision. The proposed method successfully predicted the likelihood of cervical cancer by combining scanning electron microscopy and atomic force microscopy data.

**CONCLUSION:**

The findings indicate that the triangle divergence-based multimodal classification outperforms single-modality approaches. The combined scanning electron microscopy and atomic force microscopy method using triangle divergence achieved a classification accuracy of 95.9%, demonstrating the effectiveness of this integrated approach for early cervical cancer detection.

## INTRODUCTION

Cancer is a leading global health issue and is projected to become the primary cause of death by 2030, according to WHO and IARC estimates^1^. Although diagnostic rates have improved, mortality remains high, highlighting the limitations of conventional diagnostic and treatment methods^2^. Traditional diagnostic tools, such as imaging and biopsies, often detect cancer only at advanced stages—typically when the tumor exceeds 1 cm in diameter or 1 g in mass, corresponding to approximately 100 million cells^3-5^. At this stage, treatment options become more limited and less effective. Nanotechnology offers a promising solution by enabling earlier and more precise detection. Nanostructures can target and enter individual tumor cells, significantly improving imaging sensitivity. For instance, while mammography detects tumors at the scale of a million cells, nanotechnological approaches can identify malignancies with fewer than 100 cells^6^. Studies have shown that nanoparticles conjugated with cancer-specific biomolecules can alter their physical properties in response to the cellular environment. These changes can be measured using optical, electrical, or mechanical methods, enabling early stage cancer detection^7,8^.

Such innovations represent a major advancement in the pursuit of minimally invasive, high-accuracy diagnostic techniques. Scanning electron microscopy (SEM) and atomic force microscopy (AFM) are advanced imaging techniques that provide high-resolution surface analysis. SEM uses electron beams to examine surface morphology, while AFM scans surfaces with a nanoscale probe and can operate in ambient conditions without extensive sample preparation^9-12^.

This study proposes a new method for early cervical cancer detection by combining SEM and AFM data—an approach not previously explored. The aim is to enhance diagnostic accuracy through multimodal analysis. Key features were extracted using the discrete wavelet transform (DWT). Lesion likelihoods were estimated via exponential distribution modeling.

Feature weights were calculated using triangle divergence (TD), Jensen–Shannon divergence (JSD), and Hellinger divergence (HD), and used in a decision-making algorithm to classify cells into six groups: normal, benign, or malignant (nSEM, bSEM, mSEM; nAFM, bAFM, mAFM). The combined method showed strong classification performance, with TD yielding the highest accuracy. A visual probability scale was also developed to support the interpretation of classification outcomes.

## METHODS

In this study, the cervical cell images were originally sized at 512×512 pixels. Each image was divided into four non-overlapping regions, each measuring 256×256 pixels. As a result, a total of 180 image regions were generated from 45 samples in each class (normal, benign, and malignant), with three different SEM/AFM modes, leading to 3×180 samples overall. Of these 180 image regions, 90 were used for training and the remaining 90 for testing the proposed classification algorithm. In total, 540 SEM images were used—comprising 180 normal (nSEM), 180 benign (bSEM), and 180 malignant (mSEM) samples. Similarly, 540 AFM images were obtained—180 normal (nAFM), 180 benign (bAFM), and 180 malignant (mAFM) samples^7^.

### Discrete wavelet transform

DWT has emerged as a powerful tool for feature extraction across various domains, including general signal processing, biomedical applications, and communication systems. This method involves decomposing a signal into components at different frequency bands by scaling and translating a mother wavelet over time^13,14^. Such multilevel decomposition enables both temporal and frequency-based analysis of signals or images, depending on the level of detail required. A mathematical formulation illustrating the decomposition process up to four levels using DWT is presented in Equation (1) ^15^.


(1) $$I_{BI}(u,v)=DWT\{I(x,y),B\in \{CA,CH,CV,CD\},1<I<4\}$$

### Jensen–Shannon divergence

JSD is a prominent method employed to evaluate the discrepancy between two probability distributions. In the literature, it is also referred to as the "information radius" or the "total divergence to the average." By definition, JSD is a symmetrized and smoothed version of the Kullback–Leibler Divergence (KLD). In contrast, the KLD is not symmetric—meaning the divergence from distribution t to (u DKL (t||u)) is generally not equal to the divergence from u to (t DKL (t||u)). This asymmetry ­limits KLD's usefulness in certain applications. JSD addresses this limitation by providing a symmetric distance measure between probability distributions^16,17^. The basic formulation is as follows:


(1) $$JSD_{t,u}=\sum_{c}P(t)log(\frac{P(t)}{P(u)}+(1-P(t))log(\frac{\left(1-P(t)\right)}{\left(1-P(u)\right)}$$

The DWT was implemented using the Daubechies (db4) mother wavelet with four levels of decomposition. From each level, the approximation (CA) and detail coefficients (CH, CV, CD) were analyzed. Extracted features included energy, entropy, mean, standard deviation, skewness, kurtosis, and normalized energy (NE). The NE feature was defined as:


(2) $$NE=\Sigma |C(x,y){|}^{2}/\Sigma |I(x,y){|}^{2}$$

where *C*(*x,y*) is a DWT coefficient and *I* (*x,y*) is pixel intensity. NE quantifies the proportion of total energy retained in the transformed domain, providing robustness to illumination variations.

### Hellinger divergence divergence

HD refers to the distance function between u and t. The mathematical formulation of the HD distance function is presented in Equation (3).


(3) $$HD_{t,u}=\sum_{N}(\sqrt{P(t)}-\sqrt{{\left(P(u)\right)}^{2}}$$

### Triangle divergence

TD represents the distance function between u and t. The TD distance function is provided in Equation (4).


(4) $$TD_{t,u}=\sum_{c}\frac{|P(t)-P(u){|}^{2}}{P(t)+P(u)}$$

(5) $$W_{JSDav,HDav,TDav}(i)=\sum_{j/i}\frac{\left(a_{ij}\right)}{N}JSD_{t,u},HD_{t,u},TD_{t,u}$$

(6) $$W_{JSD,HD,TD}(i)=\frac{W_{JSDavg,HDavg,TDavg}(i)}{z\sum_{j/i}P\left(a_{ij}\right)\log P\left(a_{ij}\right)}$$

where *W_JSDav, HDav, TDav_* (*i*) represents the average weight calculation and *W_JSD, HD, TD_* (*i*) denotes the weight of the extracted *i*th attribute for JSD, HD, and TD, respectively. The total number of cells is denoted by **N**, and the normalization constant is represented by **z**. The *j*th value in the *i*th attribute is *a_ij_*, while the goal attribute is **c. P(t)** represents the probabilistic value of the cells, including mSEM+mAFM, nSEM+nAFM, and bSEM+bAFM.

SEM and AFM images were obtained under controlled photodynamic illumination conditions to enhance optical contrast and improve the visualization of microstructural variations associated with cervical tissue alterations. The photodynamic setup utilized a wavelength-selective light source to excite chromophores, thereby amplifying contrast for subsequent analysis. Kernel estimation was applied to model the statistical intensity distributions across DWT sub-bands, enabling a more robust representation of feature variability and supporting the divergence-based classification.

## RESULTS

Some samples of 3×90 SEM and AFM cells are used. These 3×90 SEM and AFM cells are preferred as training cells. In this article, tests are performed by *mSEM, mAFM*, *nSEM, nAFM*, and *bSEM, bAFM* on cervical cancer cells from SEM and AFM cells. NE is one of the extracted attributes for SEM and AFM cells. *mSEM, mAFM*, *nSEM, nAFM*, and *bSEM, bAFM* limit ranges of NE attributes are obtained by utilizing the NE attribute values for 90 SEM and AFM cells. Stage I, stage II, and stage III are acquired for every attribute.

In Table 1, the *d_JSD, HD, TD_* values for normal SEM and AFM cells are between 0 and 0.4. The *d_JSD, HD, TD_* for benign SEM and AFM cells are between 0.4 and 0.6.

**Table 1:** Examples of the d-test values derived from the Jensen–Shannon divergence, Hellinger divergence, and triangle divergence classifiers for scanning electron microscopy and atomic force microscopy cells.

d-test values	d_JSD_	dHD	dTD	P(Cn\f)	P(Cb\f)	P(Cm\f)
n_AFM1_	0.23	0.19	0.12	96%	3%	1%
b_AFM1_	0.45	0.42	0.53	10%	85%	5%
m_AFM1_	0.75	0.83	0.78	2%	6%	92%
n_SEM1_	0.27	0.25	0.22	93%	4%	3%
b_SEM1_	0.55	0.50	0.48	8%	90%	2%
m_SEM1_	0.85	0.65	0.90	1%	8%	91%

n_AFM1_: normal atomic force microscopy1; b_AFM1_: benign atomic force microscopy1; m_AFM1_: malignant atomic force microscopy1; n_SEM1_: normal scanning electron microscopy1; b_SEM1_: benign scanning electron microscopy1; m_SEM1_: malignant scanning electron microscopy1.

The *d_JSD, HD, TD_* for malign SEM and AFM cell examples are found between 0.6 and 1. According to this,


$$d_{JSD,HD,TD}=\left\{\begin{array}{cc} 0-0.4, & Normal\\ 0.4-0.6, & Benign\\ 0.6-1, & Malign \end{array}\right\}$$

A total of 90 test cells for each category—nSEM, bSEM, and mSEM—are selected for SEM analysis. The *d_JSD, HD, TD_* ­values for test cell nSEM1 are 0.27, 0.25, and 0.22, respectively, as shown in Table 1. Since these values are close to 0, the cell is classified as nSEM. For test cell mSEM1, the *d_JSD, HD, TD_* values are 0.85, 0.65, and 0.90, respectively. Since these values are near 1, the cell is classified as mSEM. The *d_JSD, HD, TD_* values for test cell bSEM1 are 0.55, 0.50, and 0.48. As these values fall within the range of 0.4–0.6, the cell is classified as bSEM.

Similarly, 90 test cells each for nAFM, bAFM, and mAFM are selected for AFM analysis. For test cell nAFM1, *d_JSD, HD, TD_* values are 0.23, 0.19, and 0.12, respectively, indicating the cell is classified as nAFM due to values close to 0. For test cell mAFM1, the *d_JSD, HD, TD_* values are 0.75, 0.83, and 0.78, which are near 1, resulting in classification as mAFM. Lastly, the *d_JSD, HD, TD_* values for test bAFM1 are 0.45, 0.42, and 0.53, falling within the range of 0.4–0.6, and thus, the cell is classified as bAFM.

In this study an algorithm has been developed for cervical image classification. Classification performance of the developed system is different for every 256×256 image, which is selected from 90 cells. Correct classification performance of the developed system is given in Table 2. As seen here, the algorithm has the adaptive attribute extraction and classification tasks. The proposed study has numerous advantages with respect to other algorithms.

**Table 2:** Correct classification rate of proposed algorithm.

Type of cervical image	Correct classification rate (%)		
	JSD	HD	TD
n_AFM_	94	86	100
b_AFM_	92	96	90
m_AFM_	98	100	98
n_SEM_	96	90	94
b_SEM_	84	94	96
m_SEM_	88	96	98
The number of correct classified cervical image region	497	506	518
The total number of used cervical image region	540	540	540
Average success rate (%)	92.03	93.7	95.9

JSD: *Jensen–Shannon divergence;* HD: *Hellinger divergence;* TD: *triangle divergence;* n_AFM_: normal atomic force microscopy; b_AFM_: benign atomic force microscopy; m_AFM_: malignant atomic force microscopy; n_SEM_: normal scanning electron microscopy; b_SEM_: benign scanning electron microscopy; m_SEM_: malignant scanning electron microscopy. dJSD, dHD, and dTD are d-test values derived from the Jensen–Shannon divergence, Hellinger divergence, and triangle divergence.

One of these advantages is a high classification performance by using a small number of attributes. The performance of the proposed study for cervical image classification is given in Table 2.

In addition to overall accuracy values, statistical confidence intervals were estimated using a nonparametric bootstrap approach to ensure the robustness of classification results. Specifically, the test dataset (n=540) was resampled 10,000 times with replacement, and performance metrics—including accuracy, precision, recall, and F1-score—were recalculated for each iteration. The 95%CIs were then determined from the 2.5th and 97.5th percentiles of the resulting performance distributions. The TD classifier achieved the highest performance, with a mean accuracy of 95.90% (95%CI 94.87–96.84) and an F1-score of 0.96±0.02. The HD and JSD classifiers reached mean accuracies of 93.70% (95%CI 92.48–94.89) and 92.03% (95%CI 90.62–93.52), respectively. These results confirm the statistical significance and consistency of the proposed multimodal SEM–AFM images across resampling iterations.

## DISCUSSION

In this article, early cervical cancer diagnosis was diagnosed by SEM and AFM. In this article, TD, JSD, and HD classifiers are used. Table 2 lists the classification rates of HD, JSD, and TD. The total correct classification rate for SEM and AFM cells was found to be 92.03%.

In the second method, the HD classifier, the total correct classification rate for SEM and AFM cells is 93.7%.

In the last method, the TD classifier, the total correct classification rate for SEM and AFM cells is 95.9%.

## CONCLUSION

Many studies have been conducted in the field of cancer, yet a significant number of patients continue to succumb to the disease. To address this issue, a new approach for the early diagnosis of cervical cancer has been proposed in this study. Real medical cervical cancer cells have been utilized to conduct assessments. Approval for this research was obtained from the Ethics Committee of Clinical Investigations in Fırat University. Following approval, patient records were collected from Elazığ and other cities in Turkey. A total of 540 training cells, including 90*n_SEM_*, 90*b_SEM_*, 90*m_SEM_*, 90*n_AFM_*, 90*b_AFM_*, 90*m_AFM,_* were obtained from the Scanning Electron and Nanotechnology Laboratory at Fırat University. The objective function for these cells—*nSEM, nAFM*, *bSEM, bAFM*, *mSEM*, and *mAFM*—was established using JSD, HD, and TD measures. This article proposes a decision-making algorithm for assessing the risk of early cancer diagnosis.

Cancer risk of nSEM, nAFM, bSEM, bAFM, mSEM, and mAFM cervical cells was obtained by *d_JSD, HD, TD_* rates using TD, JSD, and HD measures, which were connected with each other. As a result, it was found that the performance of the TD classifier was higher than that of the HD and JSD classifiers.

Unlike recent deep learning approaches (CNN, transformer, or hybrid frameworks^18-25^), which require thousands of annotated images and high-end GPU resources, the proposed DWT divergence framework is lightweight and transparent. It operates on a relatively small dataset (1,080 images) without pre-training or transfer learning.

The novelty lies in **(i)** combining SEM and AFM modalities for cervical cancer classification for the first time and **(ii)** using divergence-based decision fusion (TD, JSD, HD) instead of deep neural features. This ensures interpretability and clinical adaptability in resource-limited settings.

Classification of different cells in future studies can be done using the methods suggested in this article. This study was conducted using single-center data from Fırat University, without external validation. The 256×256 patch-based approach may introduce sampling bias and limit generalizability. Future work will include multi-center collaborations and external test cohorts to confirm the robustness of the SEM–AFM multimodal pipeline. Moreover, hybrid extensions integrating divergence metrics with explainable deep learning will be explored.

## Data Availability

The datasets generated and/or analyzed during the current study are available from the corresponding author upon reasonable request.
